# Vascular Macrophages as Therapeutic Targets to Treat Intracranial Aneurysms

**DOI:** 10.3389/fimmu.2021.630381

**Published:** 2021-03-08

**Authors:** Sajjad Muhammad, Shafqat Rasul Chaudhry, Gergana Dobreva, Michael T. Lawton, Mika Niemelä, Daniel Hänggi

**Affiliations:** ^1^Department of Neurosurgery, Faculty of Medicine, Heinrich-Heine-University, Düsseldorf, Germany; ^2^Department of Neurosurgery, Helsinki University Hospital, University of Helsinki, Helsinki, Finland; ^3^Department of Anatomy and Developmental Biology, Medical Faculty Mannheim and European Center for Angioscience (ECAS), University of Heidelberg, Mannheim, Germany; ^4^Shifa College of Pharmaceutical Sciences, Shifa Tameer-e-Millat University, Islamabad, Pakistan; ^5^Department of Neurosurgery, Barrow Brain and Spine, Barrow Neurological Institute, Phoenix, AZ, United States

**Keywords:** intracranial aneurysms, monocytes, macrophages, inflammation, subarachnoid hemorrhage, stroke, macrophage polarization

## Abstract

Aneurysmal subarachnoid hemorrhage (aSAH) is a highly fatal and morbid type of hemorrhagic strokes. Intracranial aneurysms (ICAs) rupture cause subarachnoid hemorrhage. ICAs formation, growth and rupture involves cellular and molecular inflammation. Macrophages orchestrate inflammation in the wall of ICAs. Macrophages generally polarize either into classical inflammatory (M1) or alternatively-activated anti-inflammatory (M2)-phenotype. Macrophage infiltration and polarization toward M1-phenotype increases the risk of aneurysm rupture. Strategies that deplete, inhibit infiltration, ameliorate macrophage inflammation or polarize to M2-type protect against ICAs rupture. However, clinical translational data is still lacking. This review summarizes the contribution of macrophage led inflammation in the aneurysm wall and discuss pharmacological strategies to modulate the macrophageal response during ICAs formation and rupture.

## Introduction

Aneurysmal subarachnoid hemorrhage (aSAH) is a devastating subtype of hemorrhagic strokes and it accounts for 5% of all strokes. The worldwide incidence of aSAH is approximately 700000 person-years; the mortality of aSAH is approximately 40% despite appropriate surgical and medical care (1, 2). aSAH has a poor prognosis with significant lifelong morbidity and cognitive deficits for those who survive. Moreover, aSAH has a significant impact on society, as it often affects young people at the peak of their productive life (1, 2). This highly fatal and morbid type of intracranial hemorrhage is due to intracranial aneurysm (ICA) rupture in nearly 85% of SAH cases (3).

ICAs are weak ballooning, bulging, or abnormal dilatations that tend to form at arterial bifurcations due to chronic hemodynamic stress and inflammation (4). Intracranial aneurysms are usually found in 3% to 5% of the population and are slightly more prevalent among females (5). The risk factors for aneurysm development are arterial hypertension, smoking, chronic alcohol consumption, aging, female gender, and family history of aSAH in first-degree relatives (6). Some genetic disorders such as autosomal dominant polycystic kidney disease, Marfan syndrome, Ehlers-Danlos syndrome type IV, neurofibromatosis type 1, and fibromuscular dysplasia are associated with ICA formation (6). Moreover, single nucleotide gene polymorphisms (SNPs) in or near the genes *CDKN2B-AS1*, *SOX17* transcription regulator gene, endothelin receptor gene, *HDAC9*, and the gene encoding elastin have been revealed in genome-wide association studies (GWAS). Linkage analysis suggests that these genes are strongly associated with intracranial aneurysms (5). An exome-wide association study identified a SNP of the collagen type XVIIα1 chain gene to be significantly associated with aSAH (7). Most ICAs are found incidentally and need preventive care to prevent enlargement and rupture. Prevention of growth and rupture is necessary, as the current treatment modalities, such as surgical clipping and endovascular modalities (coiling, with or without stent and flow diverter placement) are associated with some risks.

Patient factors (age, sex, comorbidities, family history, previous history of SAH, hypertension and smoking) and aneurysm characteristics (size, location, wall irregularity, presence of secondary pouches) are key factors that aid in deciding upon treatment for an unruptured ICA. It is challenging to predict exactly the rupture risk based on aneurysm characteristics and patient risk factors. It is thus, unclear which ICAs require active treatment.

A better understanding of the pathobiology of ICA is important to clarify when active treatment is needed and may facilitate development of pharmacological treatments with no or minimal risk.

Recent evidence from human and animal studies revealed that macrophage-mediated cellular and molecular inflammation is the key player in aneurysm formation and rupture. Here, we briefly review the current knowledge on the role of macrophages in aneurysm formation and their rupture.

## Inflammation in Intracranial Aneurysms

The hallmarks of ICAs include endothelial cell dysfunction, smooth muscle cell phenotypic switch, matrix metalloproteinase secretion, and innate immune cell activation leading to vascular remodeling and vessel wall weakening (8–10). Histopathological analysis of aneurysm wall biopsies has revealed an upregulation of inflammatory mediators, disruption of lamina elastic interna, and thinning of media including mural cell death (11). Both cellular and molecular inflammation are crucial in aneurysm formation and rupture. Infiltration of inflammatory cells (especially macrophages) has been observed in the biopsies of ICAs, which shows a possible involvement of macrophages in aneurysm formation. NF-κB is a key transcription factor and is a major known regulator of important pro-inflammatory genes, including TNF, IL-1β, and COX-2. A genetic deletion of NF-κB has been shown to reduce ICA formation and growth (12). Moreover, pro-inflammatory genes regulated by NF-κB, including IL-1β (13), COX-2 (14), iNOS (15), and matrix metalloproteinase-9 (16) contribute to ICA formation. Furthermore, macrophage specific deletion of the prostaglandin E (PGE) receptor subtype 2 (EP2) (Ptger2), an upstream signaling receptor for NF-κB activation, significantly suppresses the development of ICAs in mice, indicating that prostaglandin E2-EP2-NF-κB signaling in macrophages plays a crucial role in ICA development (12). Intriguingly, macrophage-specific expression of a variant of IκB*α*, which abrogates the translocation of NF-κB, prevents ICA formation (17).

Transcriptomic analysis of ICAs revealed upregulation of pro-inflammatory cytokine genes associated with leukocyte infiltration (18–23). For instance, Nakaoka, Tajima (19) have shown an upregulation of genes related to inflammation, immune response and phagocytosis, whereas anti-inflammatory genes were downregulated. Similarly, upregulation of TNF-α and pro-apoptotic gene expression was shown along with suppressed IL-10 expression in ruptured ICAs. Moreover, SNPs in the IL-10 gene are associated with formation of ICAs (24, 25). Similar, transcriptomic and bioinformatic analyses of ruptured and unruptured ICAs have revealed enhanced expression and upregulation of inflammatory pathways such as TLR signaling, cytokine-cytokine receptor interaction, leukocyte trans-endothelial migration, NF-κB signaling, and many other inflammation-related gene ontology categories (18). Activation and involvement of the complement system has also been observed in ICAs and suggests that chronic inflammation underlies the pathogenesis of ICAs (10, 26). Further, shear stress due to disturbed blood flow at arterial branching points (which contributes to ICA development) upregulates inflammatory pathways such as NF-κB, promotes monocyte recruitment, and triggers sterile inflammation (27, 28). Inflammation in ICA walls is characterized by immune cell infiltration and altered composition of the immune cell populations such as natural killer cells, mast cells, lymphocytes, and importantly macrophages (29).

## Monocytes/Macrophages in Intracranial Aneurysms

Monocytes/macrophages are among the main components of innate immunity and represent important members of the mononuclear phagocyte system comprised of myeloid-derived cells (30). Data from human and animal studies has revealed an increased infiltration of immune cells in the aneurysm wall (24, 25, 31, 32). Several lines of evidence have shown increased infiltration of T and B lymphocytes and macrophages along with increased pro-inflammatory molecular expression in clinical resections of ICAs (24, 31, 32).

Studies have clearly demonstrated that monocyte/macrophage infiltration in the wall of ruptured aneurysms is not only found after aneurysmal rupture, but contributes to aneurysm formation and rupture (33). Increased monocyte/macrophage marker CD68 expression has been observed in mice carrying negative mutations of PPARγ in smooth muscle cells of cerebral arteries along with CXCL1, MCP-1, TNF-α expression upregulation. These mice have an increased incidence of aneurysm formation and rupture (34). Aoki, Frò`sen (12) demonstrated that macrophage infiltration driven by MCP-1 and activation of NF-κB involving PGE_2_-PGEP2 (PGE receptor subtype 2) signaling in the macrophages of arterial wall leads to aneurysm formation, suggesting that inflammation is not only present after aneurysm rupture, but also drives aneurysm formation. As intracranial arteries lack vasa vasorum in the arterial wall, the macrophages may infiltrate through endothelial cell junctions. Sphingosine-1-phosphate (S1P) receptor type 1 signaling activation strengthens the endothelial barrier. Interestingly, activation of S1P receptor type 1 reduced the number of infiltrated macrophages and enlargement of ICAs (35).

## Macrophage Polarization and Intracranial Aneurysms

**Figure 1:**
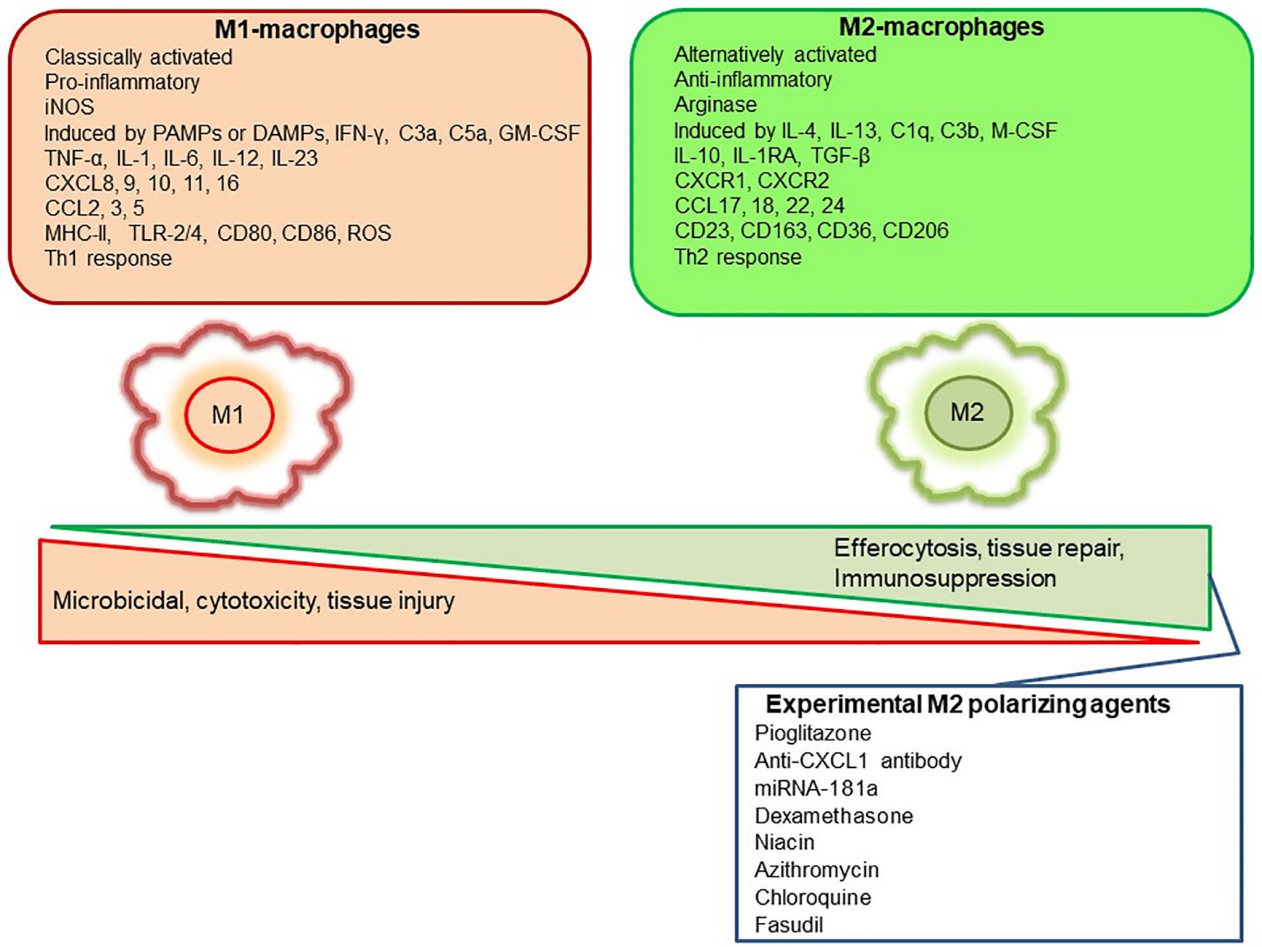
Representation of M1 and M2 distinguishing features and some of the pharmacological agents employed experimentally to polarize macrophages to M2-anti-inflammatory subtype.

**Figure 2:**
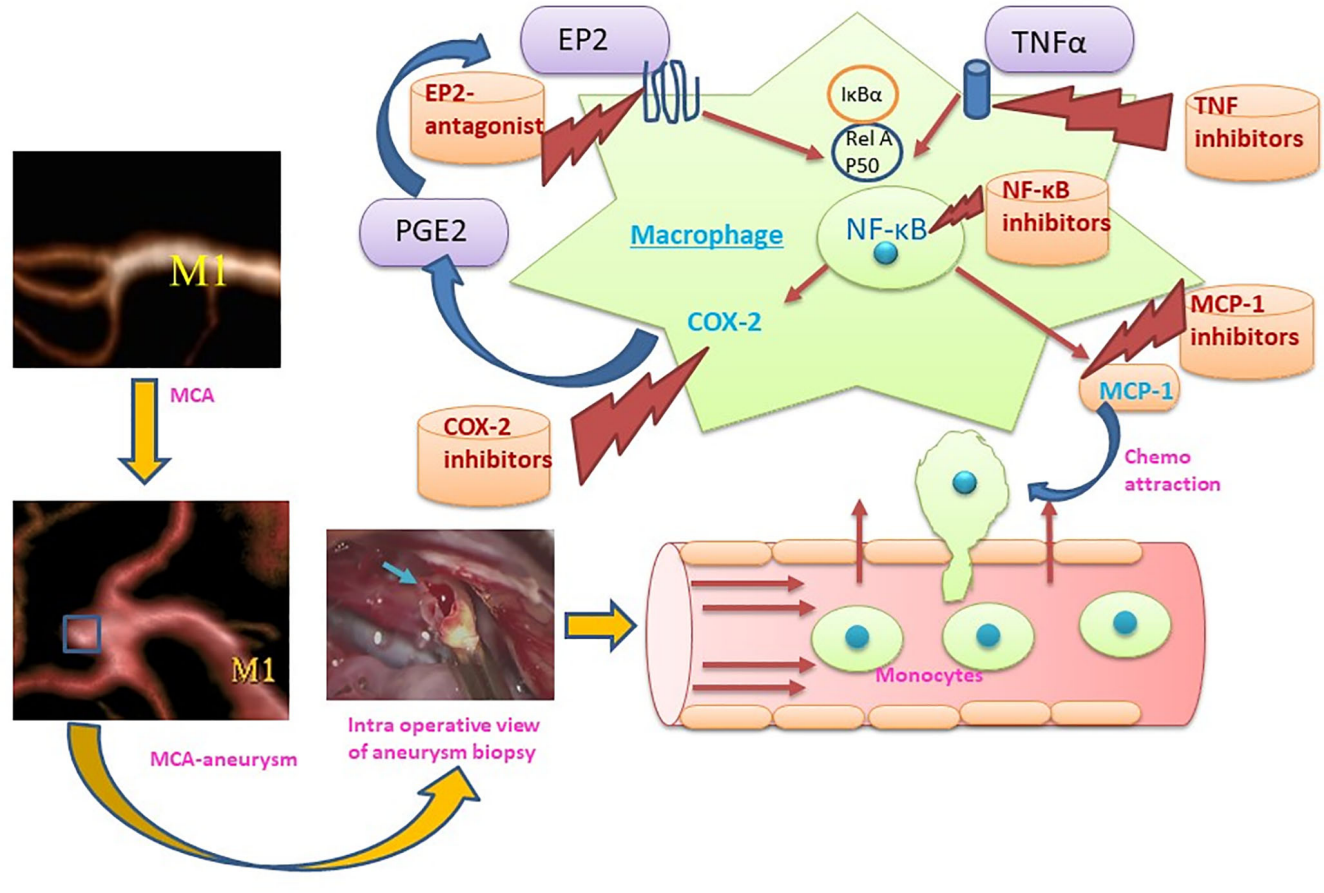
Representation of the M1 role in the formation and rupture of intracranial aneurysm and therapeutic strategies to modulate the macrophage mediated inflammation during aneurysm formation and rupture. (MCA, Middle cerebral artery: M1, M1 – Classically activated macrophages, MCP-1, Monocyte chemoattractant protein-1; COX-2, Cyclooxygenase-2, PGE2: Prostaglandin E2, EP2: Prostaglandin E2 receptor, NF-κB: nuclear factor-kappa B; TNFα, Tumor necrosis factor α).

Mills, Kincaid (36) described for the first time the M1/M2 paradigm, where M1 represents classically activated pro-inflammatory monocytes/macrophages, whereas M2 represents alternatively activated anti-inflammatory monocytes/macrophages. A very brief and simplistic overview of M1/M2 biology is represented in **Figure 1**. However, there is a considerable heterogeneity in macrophage phenotypes and several subtypes have been described such as M1, M2a, M2b, M2c, M2d, Mhem, Mox, M4 (37–40). This over simplistic representation of M1 as pro-inflammatory and M2 as anti-inflammatory macrophages is considered here to recognize different functional states of these polarized phenotypes to assign pro-inflammatory and anti-inflammatory role. Macrophage polarization has implications in aneurysm formation and rupture (**Figure 2**). Aortic aneurysms formation has been shown to be promoted by inflammatory M1 macrophages, whereas reparative M2 polarization prevents the formation, development and progression of aortic aneurysms (38, 41). It has been shown that GM-CSF contributes toward M1 polarization and M-CSF favors an M2 response (42). Intriguingly, GM-CSF has been shown to promote aortic aneurysm formation (43) and the levels of GM-CSF measured in plasma and lumen of the intracranial aneurysms have also shown a direct correlation with the size of intracranial aneurysms, highlighting a common inflammatory process upregulated by M1 macrophages underlie the development of both aortic and intracranial aneurysms (44). Consequently, immunohistochemical analysis of intracranial aneurysm dome resections have revealed that ruptured intracranial aneurysms from patients possess increased M1 (HLA-DR^+^) cells opposed to M2 (CD163^+^) cells (45). These findings suggest that a balance shift toward M2 may prevent aneurysm rupture. Previously, Froesen and colleagues demonstrated differences in CD68+ and CD163+ macrophages in human ruptured and unruptured ICAs (29). Intriguingly, CD68+ and CD163+ (hemoglobin-haptoglobin scavenger receptor) macrophages, mostly HLA-DR-, co-localize with glycophorin A (a component of the erythrocyte membrane) and infiltrate ICAs as a response to a luminal thrombus trapped and lysed erythrocytes, and may promote degenerative arterial wall remodeling (46). In a mouse model of ICAs, M1 (F4/80+ iNOS+) dominate over M2 (F4/80+ Arg1+) during aneurysm development (47). Interestingly, M1 dominance leading to aneurysm development is dependent on neutrophil infiltration, which when blocked led to an increased M2 polarization with reduced aneurysm formation (47). Shimada, Furukawa (48) employed different macrophage markers to assess the polarization of macrophages in ICAs. The authors employed CD68 as a macrophage marker and IL-12p40 and CD206 as M1 and M2 markers, respectively. They observed significant impairment in the M1/M2 ratio in ICAs associated with upregulation of M1-related gene expression (48).

## Macrophage Modulation as a Treatment Strategy for Intracranial Aneurysms

Recent case-control studies have shown that the use of statins and non-steroidal anti-inflammatory drugs (NSAIDs) is inversely associated with SAH by affecting rupture of ICAs (49–52), supporting the notion that rupture of ICAs can be prevented by pharmacological therapy.

Aspirin protects against ICA rupture through modulation of inflammatory pathways (COX-2 and microsomal PGE_2_ synthase-1 inhibition) and macrophage burden in ICAs (53–55). A prospective cohort study revealed that usage of atorvastatin in secondary prevention for ischemic stroke increases the incidence of hemorrhagic stroke (hazard ratio 1.66) (29). Therefore, statin usage to prevent aneurysm rupture should be approached with caution. Due to hemorrhagic diathesis by the antiplatelet effect, the use of NSAIDs as a pre-emptive medication to prevent SAH also requires caution. Thus, drugs with highly specific targets with minimal or no side effects should be explored for long-term prophylaxis.

Given the fact that macrophages are key players in orchestrating the inflammatory response during ICA formation and rupture, they may represent vital therapeutic targets to modulate and inhibit inflammation (48). Multiple targets at the level of macrophages and macrophage-mediated inflammation have been explored recently (**Figure 2**). Inhibition of a key chemoattractant molecule, monocyte chemotactic protein-1 (MCP-1) and depletion of macrophages are associated with reduced ICAs in animal models (8, 33) demonstrating that reducing macrophage burden with both strategies effectively prevented aneurysm formation and rupture. In contrast, CXCL1 (neutrophil chemoattractant) blockade reduced neutrophil infiltration and prevented aneurysm formation without modifying the macrophage burden in a mouse model of ICAs (47), which suggests that other molecular and cellular mechanisms may be involved. Interestingly, inhibition of neutrophil infiltration with CXCL1 inhibition is associated with a shift toward M2 macrophages from a M1 phenotype (47). Activation of PPARγ by Pioglitazone has also been shown to effectively reduce the rupture of ICAs through a reduction in infiltrating macrophages and the M1/M2 ratio (48). Clodronate liposome-mediated depletion of macrophages also reduced the rupture of ICAs similar to that shown with pioglitazone, which was associated with a decrease in M1-phenotype related gene expression (48).

In addition to cellular targets, molecular targets have also been successful in experimental models. Anagliptin, a dipeptidyl peptidase-4 inhibitor, suppresses ICA growth through inhibition of macrophage infiltration and activation *via* ERK-5-mediated suppression of NF-κB (56). Moreover, Eplerenone, a mineralocorticoid receptor blocker, has been shown to reduce ICA formation, in part *via* reduction in MCP-1, MMP-9 expression and CD68+ macrophage infiltration in a rat model of ICAs (57). A pilot clinical study showed the beneficial effects of Eplerenone in preventing growth and rupture of ICAs (58). Employment of NF-κB p50 decoy oligodeoxynucleotide (ODN) has been shown to downregulate the expression of macrophage related inflammatory genes and reduced macrophage infiltration with a decline in ICAs growth in a rat model (59). Nifedipine, the drug known to be associated with better outcomes after aSAH, has been shown to prevent the enlargement and degenerative ICAs wall changes through reduced macrophage infiltration, MCP-1, and MMP-2 expression probably by modulating the DNA binding capacity of NF-κB (60).

Macrophage polarization as a therapeutic venture has been studied across various disease models. For instance, tumor associated macrophages (TAMs) represent primarily M2 like macrophages promoting tumors and their polarization toward M1 phenotype through the application of various agents such as CSF-1R inhibitor BLZ945, anti-CSF-1 mAb, Zoledronic acid, Histidine-rich glycoprotein, Hydrazinocurcumin, vadimezan (5,6-dimethylxanthenone-4-acetic acid; DMXAA), flavone glycoside Baicalin, IL10R mAb, CD40 mAb, corosolic acid, N-(2-hydroxy acetophenone) glycinate (CuNG), imiquimod, etc. have been investigated as potential tumoricidal drugs (61, 62). Interestingly, certain pieces of evidence support the antagonism of M-CSF as a beneficial tumor therapy leading to M1 polarization of macrophages from M2 tumor associated macrophage phenotype (42). Similarly, in rheumatoid arthritis with a predominance of M1 response, an opposite approach polarizing macrophages from M1 to M2 type has been shown to reduce inflammation and disease severity (63). Several M1 to M2 polarizing agents such as gene therapy by using IL-10 DNA plasmid incorporated in nanoparticles carrying tuftsin protein to target synovial tissue macrophages, Withaferin-A incorporated in manosylated liposomes, paeoniflorin-6′-O-benzene sulfonate (CP-25), sSiglec-9, fucose/galactose analog 2-D-gal and JWH133 have been shown to reduce disease severity in experimental arthritis models and polarize macrophages to anti-inflammatory M2 type (63). Similar, approaches to modulate macrophage polarization from M1 to M2 may prevent the rupture of ICAs. For instance, molecular genetic approaches leading to polarization toward M2 macrophages using miRNAs such as miRNA-181a (64) could be of great potential. Aptamer based enrichment of M2 polarized macrophages in ICAs may also be developed (65). Epigenetic control of macrophage polarization could also be exploited to abrogate the chronic inflammation leading to ICAs formation (66). Egress of macrophages may also be promoted to decrease macrophage burden (67). Berberine, an alkaloid from *Coptis chinensis*, has been shown to inhibit macrophage activation and infiltration in ICAs by modulation of the phospho-focal adhesion kinase (pFAK)/Grp78/unfolded protein response signaling pathway and reduced the elaboration of inflammatory factors from macrophages such as MCP-1, IL-1β, IL-6, TNF-α, and MMPs (68). Intriguingly, cutaneous non-invasive vagus nerve stimulation has been shown to reduce aneurysm rupture rates and improve outcomes after aneurysm rupture, and may implicate reduced MMP-9 expression as a potential mechanism of action (69). Suppression of MMP-9 expression in macrophages and polarization of macrophages/microglia to the M2 phenotype has already been shown to stem from vagus nerve stimulation-mediated modulation of inflammatory pathways (69, 70). Taken together, there are multiple strategies at the level of inflammation and macrophage modulation that have translational potential in human disease. However, the heterogeneity and the complexity of macrophageal response should be cautiously considered in future studies aiming at characterizing the role of these main sentinel cells of ICAs inflammation (39, 40). A recent study revealed that during Ang-II induced inflammation of the aorta, primarily adventitial macrophage population expanded due to the infiltration of the bone marrow derived macrophages, whereas the residential embryonic macrophages do show local proliferation, but retain their homeostatic roles (40). Similar, studies utilizing fate mapping, mass cytometry, single cell transcriptomics and proteomics may be required to unveil the complexity and heterogeneity of ICAs macrophages, which may be helpful to design better therapeutic strategies. A summary of various clinical and preclinical observations and interventions aiming to prevent macrophage mediated inflammation in ICAs is represented in **Table 1**. Clinical trials are needed to confirm the efficacy observed in animal studies.

**Table 1:** A brief summary of macrophage modulation studies for prevention of intracranial aneurysms (ICAs) formation and rupture.

Study Type	Model	Intervention	Macrophage markers	Main Findings	References
Clinical	–	No intervention	CD68+, CD163+, HLA-DR+	CD68+, CD163+, HLA-DR- macrophages infiltrate ICAs and correlate with GPA^f^, loss of α-SMA, wall degeneration, rupture	Ollikainen et al. (46)
Clinical	–	No intervention	CD68+, CD163+, CD11b+	CD68+, CD163+, CD11b+ macrophages increased in ruptured than unruptured ICAs	Froesen et al. (29)
Clinical	–	No intervention	HLA-DR+ (M1), CD163+ (M2)	M1 macrophages were dominant compared to M2 macrophages in ruptured ICAs	Hasan et al. (45)
Clinical	–	ferumoxytol enhanced MRI	CD68+	Increased macrophage infiltration in ICAs wall assessed by enhanced uptake of ferumoxytol and CD68+ expression	Hasan et al. (45)
Clinical	–	Aspirin 81 mg for 3 months, ferumoxytol enhanced MRI		Decreased inflammation in ICAs due to macrophages with daily intake of Aspirin	Hasan et al. (54)
Preclinical	C57BL/6J mice, Elastase & Ang.II^g^ induced HTN^c^	Clodronate MCP-1 KO^a^, MMP-12 KO	CD68+	Increased CD68+ macrophage infiltration in ICAs, macrophage depletion and MCP-1 KO reduced ICAs formation	Kanematsu et al. (33)
Preclinical	Male Sprague Dawley rats, left internal carotid artery ligation, elastase and high salt diet	Berberine 200mg/kg/d for 35 days	CD68+	CD68+ macrophages infiltration in ICAs was decreased by berberine through suppressed expression of MMP-9 and secretion of MCP-1, IL-1β, TNF-α, and IL-6 *via* down regulation of pFAK/Grp78/UPR signaling pathway	Quan et al. (68)
Preclinical	C57BL/6J mice, ligation of left CCA and right renal artery, Ang. II, elastase, 8% NaCl, 0.12% β-aminopropionitrile	anti-CXCL1/GRO-α/KC/CINC-1 antibody	F4/80+, iNOS+ (M1), Arg1+ (M2)	M1/M2 ratio increased in ICAs formation over time, CXCL1 blocked of neutrophils shifted the polarization toward M2 macrophages and reduced aneurysm formation	Nowicki et al. (47)
Preclinical	Deoxycorticosterone acetate-salt HTN, elastase	Pioglitazone 10 mg/kg/d^b^, GW9662 2 mg/kg/d for 3 weeks, macrophage PPR*γ* KO, clodronate liposome depletion of macrophages	CD68+, IL-12 p40 (M1), CD206 (M2), CD36	Pioglitazone reduced the incidence and rupture of ICAs *via* reduced infiltration of M1 and M1/M2 ratio in cerebral arteries. Pioglitazone effect was lost in macrophage specific PPR*γ* KO. Pioglitazone also reduced expression of MCP-1, IL-1 and IL-6.	Shimada et al. (48)
Preclinical	Male Sprague Dawley rats, left renal and common carotid arteries ligation, 8% sodium chloride and 0.12% 3‐aminopropionitrile,	Anagliptin 300 mg/kg	Iba-1+, MCP-1+	Anagliptin prevented the growth of ICAs, inhibited the infiltration and activation of macrophages through reduced MCP-1 expression and suppressed p65 phosphorylation through ERK5 activation.	Ikedo et al. (56)
Preclinical	Female Sprague-Dawley rats, Ligation of right common carotid artery and renal artery, 1% saline administration and bilateral oophorectomy	Eplerenone30 or 100 mg/kg/d	CD68+, MCP-1+	Increased infiltration of CD68+ macrophages in ICAs walls with upregulation of MCP-1 and MMP-9, which was prevented by Eplerenone administration associated with reduced incidence of ICAs.	Tada et al. (57)
Preclinical	Sprague-Dawley rats, unilateral ligation of common carotid artery along with β-aminopropionitrile	NF-κB p50 KO, NF-κB decoy ODN^d^ 40 μg/60 μl every 2 weeks	CD68+, MCP-1+	Activated NF-κB (p65) colocalized with CD68+ macrophages in ICAs and also with MCP-1 and VCAM-1. Gene expression of MCP-1, VCAM-1, MMP-2, MMP-9, IL-1β, and iNOS along with reduced infiltration of CD68+ macrophages was observed in NF-κB p50 KO mice associated with decreased incidence of ICAs formation. Macrophage infiltration, expression of downstream genes, and ICAs formation were dramatically inhibited by NF-κB decoy ODN.	Aoki et al. (16)
Preclinical	Sprague-Dawley rats, ligation of left common carotid artery and posterior branches of bilateral renal arteries, 8% sodium chloride and 0.12% β-aminopropionitrile	Nifedipine 10mg/kg/d for 2 months i.p^e^.	CD68+, MCP-1+	Nifedipine prevented the enlargement and degeneration of the walls of preexisting ICAs. Nifedinpine led to reduced macrophage infiltration, MCP-1, MMP-2 expression and NF-κB DNA binding.	Aoki et al. (60)
Preclinical	Sprague-Dawley rats C57BL/6NCrSlc, Ligation of the left common carotid artery and left renal artery along with a salt loading dose (8% 0.12% 3-aminopropionitrile	Macrophage-specific deletion of Ptger2 (which encodes EP2) or macrophage-specific expression of an IκBα mutant that restricts NF-κB activation, EP2 antagonist	CD68+	EP2 and COX-2 correlated with ICAs macrophage infiltration. NF-κB activated in macrophages in the adventitia and in endothelial cells and, subsequently, in the entire arterial wall. Upregulation of proinflammatory genes, including Ptgs2 (encoding COX-2). EP2 signaling also stabilized CCL2 (encoding MCP-1). Rats administered an EP2 antagonist had reduced macrophage infiltration and ICAs formation and progression	Aoki et al. (12)

^a^KO, Knock out; ^b^d, day; ^c^HTN, Hypertension; ^d^ODN, oligodeoxynucleotide; ^e^i.p, intraperitoneally; ^f^GPA, Glycophorin A; ^g^Ang. II, Angiotensin II.

## Conclusion

Inflammation and macrophages represent the cornerstones of ICAs development and rupture. Macrophage modulation seems to represent an important therapeutic target and may lead to treatments against ICAs growth and rupture.

## Author Contributions

SM conceived the idea, contributed to the initial manuscript draft, and reviewed and edited it. SC significantly contributed to the initial manuscript draft. GD, ML, MN, and DH critically reviewed the final draft of the manuscript for the intellectual content. All authors contributed to the article and approved the submitted version.

## Conflict of Interest

The authors declare that the research was conducted in the absence of any commercial or financial relationships that could be construed as a potential conflict of interest.

## References

[1] Hackenberg Katharina AM Hänggi D Etminan N. Unruptured Intracranial Aneurysms. Stroke (2018) 49(9):2268–75. 10.1161/STROKEAHA.118.021030 30355003

[2] Macdonald RL. Delayed neurological deterioration after subarachnoid haemorrhage. Nat Rev Neurol (2014) 10(1):44–58. 10.1038/nrneurol.2013.246 24323051

[3] Macdonald RL Schweizer TA. Spontaneous subarachnoid haemorrhage. Lancet (2017) 389(10069):655–66. 10.1016/S0140-6736(16)30668-7 27637674

[4] Lawton MT Vates GE. Subarachnoid Hemorrhage. New Engl J Med (2017) 377(3):257–66. 10.1056/NEJMcp1605827 28723321

[5] Etminan N Rinkel GJ. Unruptured intracranial aneurysms: development, rupture and preventive management. Nat Rev Neurol (2016) 12(12):699–713. 10.1038/nrneurol.2016.150 27808265

[6] D’Souza S. Aneurysmal Subarachnoid Hemorrhage. J Neurosurg Anesthesiol (2015) 27(3):222–40. 10.1097/ANA.0000000000000130 PMC4463029 25272066

[7] Yamada Y Sakuma J Takeuchi I Yasukochi Y Kato K Oguri M. Identification of six polymorphisms as novel susceptibility loci for ischemic or hemorrhagic stroke by exome-wide association studies. Int J Mol Med (2017) 39(6):1477–91. 10.3892/ijmm.2017.2972 PMC5428971 28487959

[8] Aoki T Kataoka H Ishibashi R Nozaki K Egashira K Hashimoto N. Impact of monocyte chemoattractant protein-1 deficiency on cerebral aneurysm formation. Stroke (2009) 40(3):942–51. 10.1161/STROKEAHA.108.532556 19164781

[9] Strong MJ Amenta PS Dumont AS Medel R. The role of leukocytes in the formation and rupture of intracranial aneurysms. Neuroimmunol Neuroinflamm (2015) 2:107–14. 10.4103/2347-8659.153972

[10] Tulamo R Frosen J Junnikkala S Paetau A Kangasniemi M Pelaez J. Complement system becomes activated by the classical pathway in intracranial aneurysm walls. Lab Invest (2010) 90(2):168–79. 10.1038/labinvest.2009.133 19997064

[11] Frosen J Tulamo R Paetau A Laaksamo E Korja M Laakso A. Saccular intracranial aneurysm: pathology and mechanisms. Acta Neuropathol (2012) 123(6):773–86. 10.1007/s00401-011-0939-3 22249619

[12] Aoki T Frò`sen J Fukuda M Bando K Shioi G Tsuji K. Prostaglandin E_2_–EP2–NF-κB signaling in macrophages as a potential therapeutic target for intracranial aneurysms. Sci Signaling (2017) 10(465):eaah6037. 10.1126/scisignal.aah6037 28174280

[13] Moriwaki T Takagi Y Sadamasa N Aoki T Nozaki K Hashimoto N. Impaired progression of cerebral aneurysms in interleukin-1beta-deficient mice. Stroke (2006) 37(3):900–5. 10.1161/01.STR.0000204028.39783.d9 16439700

[14] Aoki T Nishimura M Matsuoka T Yamamoto K Furuyashiki T Kataoka H. PGE2-EP2 signalling in endothelium is activated by haemodynamic stress and induces cerebral aneurysm through an amplifying loop via NF-κB. Br J Pharmacol (2011) 163(6):1237–49. 10.1111/j.1476-5381.2011.01358.x PMC3144537 21426319

[15] Sadamasa N Nozaki K Hashimoto N. Disruption of gene for inducible nitric oxide synthase reduces progression of cerebral aneurysms. Stroke (2003) 34(12):2980–4. 10.1161/01.str.0000102556.55600.3b 14615616

[16] Aoki T Kataoka H Morimoto M Nozaki K Hashimoto N. Macrophage-derived matrix metalloproteinase-2 and -9 promote the progression of cerebral aneurysms in rats. Stroke (2007) 38(1):162–9. 10.1161/01.STR.0000252129.18605.c8 17122420

[17] Shimizu K Kushamae M Mizutani T Aoki T. Intracranial Aneurysm as a Macrophage-mediated Inflammatory Disease. Neurol Med-Chirurg (2019) 59(4):126–32. 10.2176/nmc.st.2018-0326 PMC6465529 30867357

[18] Kurki MI Hakkinen SK Frosen J Tulamo R von und zu Fraunberg M Wong G. Upregulated signaling pathways in ruptured human saccular intracranial aneurysm wall: an emerging regulative role of Toll-like receptor signaling and nuclear factor-kappaB, hypoxia-inducible factor-1A, and ETS transcription factors. Neurosurgery (2011) 68(6):1667–75; discussion 75-6. 10.1227/NEU.0b013e318210f001 21336216

[19] Nakaoka H Tajima A Yoneyama T Hosomichi K Kasuya H Mizutani T. Gene expression profiling reveals distinct molecular signatures associated with the rupture of intracranial aneurysm. Stroke (2014) 45(8):2239–45. 10.1161/strokeaha.114.005851 24938844

[20] Pera J Korostynski M Krzyszkowski T Czopek J Slowik A Dziedzic T. Gene expression profiles in human ruptured and unruptured intracranial aneurysms: what is the role of inflammation? Stroke (2010) 41(2):224–31. 10.1161/strokeaha.109.562009 20044533

[21] Shi C Awad IA Jafari N Lin S Du P Hage ZA. Genomics of human intracranial aneurysm wall. Stroke (2009) 40(4):1252–61. 10.1161/strokeaha.108.532036 19228845

[22] Weinsheimer S Lenk GM van der Voet M Land S Ronkainen A Alafuzoff I. Integration of expression profiles and genetic mapping data to identify candidate genes in intracranial aneurysm. Physiol Genomics (2007) 32(1):45–57. 10.1152/physiolgenomics.00015.2007 17878320

[23] Krischek B Kasuya H Tajima A Akagawa H Sasaki T Yoneyama T. Network-based gene expression analysis of intracranial aneurysm tissue reveals role of antigen presenting cells. Neuroscience (2008) 154(4):1398–407. 10.1016/j.neuroscience.2008.04.049 18538937

[24] Jayaraman T Berenstein V Li X Mayer J Silane M Shin YS. Tumor Necrosis Factor α is a Key Modulator of Inflammation in Cerebral Aneurysms. Neurosurgery (2005) 57(3):558–64. 10.1227/01.NEU.0000170439.89041.D6 16145536

[25] Sathyan S Koshy LV Srinivas L Easwer HV Premkumar S Nair S. Pathogenesis of intracranial aneurysm is mediated by proinflammatory cytokine TNFA and IFNG and through stochastic regulation of IL10 and TGFB1 by comorbid factors. J Neuroinflamm (2015) 12(1):1–10. 10.1186/s12974-015-0354-0 PMC4510902 26198819

[26] Tulamo R Frosen J Junnikkala S Paetau A Pitkaniemi J Kangasniemi M. Complement activation associates with saccular cerebral artery aneurysm wall degeneration and rupture. Neurosurgery (2006) 59(5):1069–76; discussion 76-7. Epub 2006/10/04. 10.1227/01.NEU.0000245598.84698.26 17016232

[27] Baeriswyl DC Prionisti I Peach T Tsolkas G Chooi KY Vardakis J. Disturbed flow induces a sustained, stochastic NF-κB activation which may support intracranial aneurysm growth in vivo. Sci Rep (2019) 9(1):4738. 10.1038/s41598-019-40959-y 30894565 PMC6426999

[28] Cuhlmann S Van der Heiden K Saliba D Tremoleda JL Khalil M Zakkar M. Disturbed blood flow induces RelA expression via c-Jun N-terminal kinase 1: a novel mode of NF-kappaB regulation that promotes arterial inflammation. Circ Res (2011) 108(8):950–9. 10.1161/circresaha.110.233841 21350211

[29] Frosen J Piippo A Paetau A Kangasniemi M Niemela M Hernesniemi J. Remodeling of saccular cerebral artery aneurysm wall is associated with rupture: histological analysis of 24 unruptured and 42 ruptured cases. Stroke (2004) 35(10):2287–93. 10.1161/01.STR.0000140636.30204.da 15322297

[30] Jakubzick CV Randolph GJ Henson PM. Monocyte differentiation and antigen-presenting functions. Nat Rev Immunol (2017) 17(6):349–62. 10.1038/nri.2017.28 28436425

[31] Kataoka K Taneda M Asai T Kinoshita A Ito M Kuroda R. Structural fragility and inflammatory response of ruptured cerebral aneurysms. A comparative study between ruptured and unruptured cerebral aneurysms. Stroke (1999) 30(7):1396–401. 10.1161/01.str.30.7.1396 10390313

[32] Chyatte D Bruno G Desai S Todor DR. Inflammation and intracranial aneurysms. Neurosurgery (1999) 45(5):1137–46; discussion 46-7. 10.1097/00006123-199911000-00024 10549930

[33] Kanematsu Y Kanematsu M Kurihara C Tada Y Tsou TL van Rooijen N. Critical roles of macrophages in the formation of intracranial aneurysm. Stroke (2011) 42(1):173–8. 10.1161/strokeaha.110.590976 PMC3021554 21106959

[34] Hasan DM Starke RM Gu H Wilson K Chu Y Chalouhi N. Smooth Muscle Peroxisome Proliferator-Activated Receptor gamma Plays a Critical Role in Formation and Rupture of Cerebral Aneurysms in Mice In Vivo. Hypertens (Dallas Tex 1979) (2015) 66(1):211–20. 10.1161/hypertensionaha.115.05332 PMC4465866 25916724

[35] Yamamoto R Aoki T Koseki H Fukuda M Hirose J Tsuji K. A sphingosine-1-phosphate receptor type 1 agonist, ASP4058, suppresses intracranial aneurysm through promoting endothelial integrity and blocking macrophage transmigration. Br J Pharmacol (2017) 174(13):2085–101. 10.1111/bph.13820 PMC5466536 28409823

[36] Mills CD Kincaid K Alt JM Heilman MJ Hill AM. M-1/M-2 macrophages and the Th1/Th2 paradigm. J Immunol (2000) 164(12):6166–73. 10.4049/jimmunol.164.12.6166 10843666

[37] Boyle JJ Johns M Kampfer T Nguyen AT Game L Schaer DJ. Activating transcription factor 1 directs Mhem atheroprotective macrophages through coordinated iron handling and foam cell protection. Circ Res (2012) 110(1):20–33. 10.1161/circresaha.111.247577 22052915

[38] Cheng Z Zhou Y-Z Wu Y Wu Q-Y Liao X-B Fu X-M. Diverse roles of macrophage polarization in aortic aneurysm: destruction and repair. J Trans Med (2018) 16(1):354. 10.1186/s12967-018-1731-0 PMC6293547 30545380

[39] Murray PJ Allen JE Biswas SK Fisher EA Gilroy DW Goerdt S. Macrophage activation and polarization: nomenclature and experimental guidelines. Immunity (2014) 41(1):14–20. 10.1016/j.immuni.2014.06.008 25035950 PMC4123412

[40] Weinberger T Esfandyari D Messerer D Percin G Schleifer C Thaler R. Ontogeny of arterial macrophages defines their functions in homeostasis and inflammation. Nat Commun (2020) 11(1):4549. 10.1038/s41467-020-18287-x 32917889 PMC7486394

[41] Li H Bai S Ao Q Wang X Tian X Li X. Modulation of Immune-Inflammatory Responses in Abdominal Aortic Aneurysm: Emerging Molecular Targets. J Immunol Res (2018) 2018:7213760. 10.1155/2018/7213760 29967801 PMC6008668

[42] Hamilton TA Zhao C Pavicic PG Datta S. Myeloid Colony-Stimulating Factors as Regulators of Macrophage Polarization. Front Immunol (2014) 5:554. 10.3389/fimmu.2014.00554 25484881 PMC4240161

[43] Ye P Chen W Wu J Huang X Li J Wang S. GM-CSF contributes to aortic aneurysms resulting from SMAD3 deficiency. J Clin Invest (2013) 123(5):2317–31. 10.1172/JCI67356 PMC3635740 23585475

[44] Chalouhi N Theofanis T Starke RM Zanaty M Jabbour P Dooley SA. Potential role of granulocyte-monocyte colony-stimulating factor in the progression of intracranial aneurysms. DNA Cell Biol (2015) 34(1):78–81. 10.1089/dna.2014.2618 25389911 PMC4281873

[45] Hasan D Chalouhi N Jabbour P Hashimoto T. Macrophage imbalance (M1 vs. M2) and upregulation of mast cells in wall of ruptured human cerebral aneurysms: preliminary results. J Neuroinflamm (2012) 9(1):1–7. 10.1186/1742-2094-9-222 PMC3488554 22999528

[46] Ollikainen E Tulamo R Kaitainen S Honkanen P Lehti S Liimatainen T. Macrophage Infiltration in the Saccular Intracranial Aneurysm Wall as a Response to Locally Lysed Erythrocytes That Promote Degeneration. J Neuropathol Exp Neurol (2018) 77(10):890–903. 10.1093/jnen/nly068 30113655

[47] Nowicki KW Hosaka K Walch FJ Scott EW Hoh BL. M1 macrophages are required for murine cerebral aneurysm formation. J Neurointervent Surg (2018) 10(1):93–7. 10.1136/neurintsurg-2016-012911 PMC7814362 28196918

[48] Shimada K Furukawa H Wada K Korai M Wei Y Tada Y. Protective Role of Peroxisome Proliferator-Activated Receptor-gamma in the Development of Intracranial Aneurysm Rupture. Stroke (2015) 46(6):1664–72. 10.1161/strokeaha.114.007722 PMC4447500 25931465

[49] Hasan DM Mahaney KB Brown RD Jr Meissner I Piepgras DG Huston J. Aspirin as a promising agent for decreasing incidence of cerebral aneurysm rupture. Stroke (2011) 42(11):3156–62. 10.1161/STROKEAHA.111.619411 PMC3432499 21980208

[50] Can A Castro VM Dligach D Finan S Yu S Gainer V. Lipid-Lowering Agents and High HDL (High-Density Lipoprotein) Are Inversely Associated With Intracranial Aneurysm Rupture. Stroke (2018) 49(5):1148–54. 10.1161/strokeaha.117.019972 PMC5915939 29622625

[51] Yoshimura Y Murakami Y Saitoh M Yokoi T Aoki T Miura K. Statin Use and Risk of Cerebral Aneurysm Rupture: A Hospital-based Case–control Study in Japan. J Stroke Cerebrovasc Dis (2014) 23(2):343–8. 10.1016/j.jstrokecerebrovasdis.2013.04.022 23697760

[52] Can A Rudy RF Castro VM Yu S Dligach D Finan S. Association between aspirin dose and subarachnoid hemorrhage from saccular aneurysms: A case-control study. Neurology (2018) 91(12):e1175–81. 10.1212/wnl.0000000000006200 PMC6161553 30135253

[53] Chalouhi N Atallah E Jabbour P Patel PD Starke RM Hasan D. Aspirin for the Prevention of Intracranial Aneurysm Rupture. Neurosurgery (2017) 64(CN_suppl_1):114–8. 10.1093/neuros/nyx299 28899056

[54] Hasan DM Chalouhi N Jabbour P Dumont AS Kung DK Magnotta VA. Evidence that acetylsalicylic acid attenuates inflammation in the walls of human cerebral aneurysms: preliminary results. J Am Heart Assoc (2013) 2(1):e000019. 10.1161/jaha.112.000019 23525414 PMC3603234

[55] Hasan DM Chalouhi N Jabbour P Magnotta VA Kung DK Young WL. Imaging aspirin effect on macrophages in the wall of human cerebral aneurysms using ferumoxytol-enhanced MRI: preliminary results. J Neuroradiol J Neuroradiol (2013) 40(3):187–91. 10.1016/j.neurad.2012.09.002 PMC7853080 23428244

[56] Ikedo T Minami M Kataoka H Hayashi K Nagata M Fujikawa R. Dipeptidyl Peptidase-4 Inhibitor Anagliptin Prevents Intracranial Aneurysm Growth by Suppressing Macrophage Infiltration and Activation. J Am Heart Assoc (2017) 6(6):e004777. 10.1161/jaha.116.004777 28630262 PMC5669147

[57] Tada Y Kitazato KT Tamura T Yagi K Shimada K Kinouchi T. Role of Mineralocorticoid Receptor on Experimental Cerebral Aneurysms in Rats. Hypertens (Dallas Tex 1979) (2009) 54(3):552–7. 10.1161/HYPERTENSIONAHA.109.134130 19620512

[58] Nagahiro S Tada Y Satomi J Kinouchi T Kuwayama K Yagi K. Treatment of Unruptured Cerebral Aneurysms with the Mineralocorticoid Receptor Blocker Eplerenone—Pilot Study. J Stroke Cerebrovasc Dis (2018) 27(8):2134–40. 10.1016/j.jstrokecerebrovasdis.2018.03.008 29622372

[59] Aoki T Kataoka H Shimamura M Nakagami H Wakayama K Moriwaki T. NF-κB Is a Key Mediator of Cerebral Aneurysm Formation. Circulation (2007) 116(24):2830–40. 10.1161/CIRCULATIONAHA.107.728303 18025535

[60] Aoki T Kataoka H Ishibashi R Nozaki K Hashimoto N. Nifedipine inhibits the progression of an experimentally induced cerebral aneurysm in rats with associated down-regulation of NF-kappa B transcriptional activity. Curr Neurovasc Res (2008) 5(1):37–45. 10.2174/156720208783565663 18289020

[61] Zheng X Turkowski K Mora J Brüne B Seeger W Weigert A. Redirecting TAMs to become tumoricidal effectors as a novel strategy for cancer therapy. Oncotarget (2017) 8(29):48436–52. 10.18632/oncotarget.17061 PMC5564660 28467800

[62] Genard G Lucas S Michiels C. Reprogramming of Tumor-Associated Macrophages with Anticancer Therapies: Radiotherapy versus Chemo- and Immunotherapies. Front Immunol (2017) 8:828. 10.3389/fimmu.2017.00828 28769933 PMC5509958

[63] Tardito S Martinelli G Soldano S Paolino S Pacini G Patane M. Macrophage M1/M2 polarization and rheumatoid arthritis: A systematic review. Autoimmun Rev (2019) 18(11):102397. 10.1016/j.autrev.2019.102397 31520798

[64] Bi J Zeng X Zhao L Wei Q Yu L Wang X. miR-181a Induces Macrophage Polarized to M2 Phenotype and Promotes M2 Macrophage-mediated Tumor Cell Metastasis by Targeting KLF6 and C/EBPalpha. Mol Ther Nucleic Acids (2016) 5(9):e368. 10.1038/mtna.2016.71 27673564 PMC5056994

[65] Enam SF Bellamkonda RV. FKN-aptamer functionalized hydrogels for local enrichment of M2 macrophages after traumatic brain injury. Front Bioeng Biotechnol. (2016) 4. 10.3389/conf.FBIOE.2016.01.00132

[66] de Groot AE Pienta KJ. Epigenetic control of macrophage polarization: implications for targeting tumor-associated macrophages. Oncotarget (2018) 9(29):20908–27. 10.18632/oncotarget.24556 PMC5945509 29755698

[67] Randolph GJ. Mechanisms that regulate macrophage burden in atherosclerosis. Circ Res (2014) 114(11):1757–71. 10.1161/circresaha.114.301174 PMC4059102 24855200

[68] Quan K Li S Wang D Shi Y Yang Z Song J. Berberine Attenuates Macrophages Infiltration in Intracranial Aneurysms Potentially Through FAK/Grp78/UPR Axis. Front Pharmacol (2018) 9:565. 10.3389/fphar.2018.00565 29899701 PMC5988844

[69] Suzuki T Takizawa T Kamio Y Qin T Hashimoto T Fujii Y. Noninvasive Vagus Nerve Stimulation Prevents Ruptures and Improves Outcomes in a Model of Intracranial Aneurysm in Mice. Stroke (2019) 50(5):Strokeaha118023928. 10.1161/strokeaha.118.023928 PMC6476688 30943885

[70] Zhao XP Zhao Y Qin XY Wan LY Fan XX. Non-invasive Vagus Nerve Stimulation Protects Against Cerebral Ischemia/Reperfusion Injury and Promotes Microglial M2 Polarization Via Interleukin-17A Inhibition. J Mol Neurosci MN (2019) 67(2):217–26. 10.1007/s12031-018-1227-7 30484061

